# Supplementary material for: Time-series transcriptome analysis identified differentially expressed genes in broiler chicken infected with mixed Eimeria species
Source: Front Genet. 2022 Aug 8;13:886781. doi: 10.3389/fgene.2022.886781 (PMC9393255; doi:10.3389/fgene.2022.886781)
Supplement: Supplementary file 2 [file DataSheet1.ZIP › 7dpi_KEGG.Gsea.1625115961950/KEGG_RIBOSOME.html]

Details for gene set KEGG\_RIBOSOME[GSEA]

|  || Dataset | TMM\_7dpi\_gct\_format\_7dpi\_gct\_format.Class\_4dpi.cls #PC\_versus\_NC.Class\_4dpi.cls #PC\_versus\_NC\_repos |
| Phenotype | Class\_4dpi.cls#PC\_versus\_NC\_repos |
| Upregulated in class | 1 |
| GeneSet | KEGG\_RIBOSOME |
| Enrichment Score (ES) | 0.5437216 |
| Normalized Enrichment Score (NES) | 2.5979238 |
| Nominal p-value | 0.0 |
| FDR q-value | 0.0 |
| FWER p-Value | 0.0 |
Table: GSEA Results Summary

  

Fig 1: Enrichment plot: KEGG\_RIBOSOME      
 Profile of the Running ES Score & Positions of GeneSet Members on the Rank Ordered List

  

| SYMBOL | TITLE | RANK IN GENE LIST | RANK METRIC SCORE | RUNNING ES | CORE ENRICHMENT || 1 | RPS28 | na | 529 | 0.721 | 0.0081 | Yes |
| 2 | RPS29 | na | 740 | 0.617 | 0.0354 | Yes |
| 3 | RPS27L | na | 833 | 0.579 | 0.0698 | Yes |
| 4 | RPL26L1 | na | 1025 | 0.512 | 0.0911 | Yes |
| 5 | UBA52 | na | 1369 | 0.413 | 0.0924 | Yes |
| 6 | RPS27 | na | 1424 | 0.399 | 0.1169 | Yes |
| 7 | RPS19 | na | 1540 | 0.368 | 0.1340 | Yes |
| 8 | RPL38 | na | 1576 | 0.361 | 0.1573 | Yes |
| 9 | RPL36 | na | 1577 | 0.361 | 0.1835 | Yes |
| 10 | RPL23A | na | 1779 | 0.313 | 0.1894 | Yes |
| 11 | RPL29 | na | 1780 | 0.313 | 0.2122 | Yes |
| 12 | RPS15 | na | 1804 | 0.306 | 0.2325 | Yes |
| 13 | RPLP1 | na | 1813 | 0.304 | 0.2539 | Yes |
| 14 | RPL36A | na | 1833 | 0.301 | 0.2742 | Yes |
| 15 | RPL19 | na | 1834 | 0.300 | 0.2961 | Yes |
| 16 | RPL37A | na | 1983 | 0.274 | 0.3036 | Yes |
| 17 | MRPL13 | na | 2109 | 0.251 | 0.3114 | Yes |
| 18 | RPL22 | na | 2201 | 0.237 | 0.3210 | Yes |
| 19 | RPL35A | na | 2241 | 0.231 | 0.3345 | Yes |
| 20 | RSL24D1 | na | 2281 | 0.225 | 0.3476 | Yes |
| 21 | RPL34 | na | 2286 | 0.224 | 0.3635 | Yes |
| 22 | RPL27 | na | 2300 | 0.222 | 0.3786 | Yes |
| 23 | RPS26 | na | 2332 | 0.216 | 0.3917 | Yes |
| 24 | RPL14 | na | 2343 | 0.215 | 0.4065 | Yes |
| 25 | RPS24 | na | 2347 | 0.214 | 0.4219 | Yes |
| 26 | RPL23 | na | 2383 | 0.209 | 0.4342 | Yes |
| 27 | RPS7 | na | 2413 | 0.204 | 0.4466 | Yes |
| 28 | RPL7 | na | 2670 | 0.168 | 0.4373 | Yes |
| 29 | RPL31 | na | 2693 | 0.164 | 0.4474 | Yes |
| 30 | RPL24 | na | 2734 | 0.160 | 0.4557 | Yes |
| 31 | RPS8 | na | 2771 | 0.154 | 0.4639 | Yes |
| 32 | RPL21 | na | 2810 | 0.149 | 0.4716 | Yes |
| 33 | RPL32 | na | 2870 | 0.142 | 0.4770 | Yes |
| 34 | RPL13 | na | 2902 | 0.138 | 0.4844 | Yes |
| 35 | RPS17 | na | 2988 | 0.128 | 0.4866 | Yes |
| 36 | RPS15A | na | 3019 | 0.124 | 0.4931 | Yes |
| 37 | RPS13 | na | 3028 | 0.122 | 0.5013 | Yes |
| 38 | RPLP2 | na | 3040 | 0.121 | 0.5092 | Yes |
| 39 | RPL6 | na | 3048 | 0.121 | 0.5174 | Yes |
| 40 | RPL27A | na | 3078 | 0.116 | 0.5234 | Yes |
| 41 | RPS20 | na | 3085 | 0.115 | 0.5312 | Yes |
| 42 | RPL9 | na | 3146 | 0.107 | 0.5340 | Yes |
| 43 | RPS25 | na | 3324 | 0.084 | 0.5253 | Yes |
| 44 | RPL30 | na | 3365 | 0.078 | 0.5276 | Yes |
| 45 | FAU | na | 3367 | 0.078 | 0.5332 | Yes |
| 46 | RPS10 | na | 3396 | 0.074 | 0.5363 | Yes |
| 47 | RPL11 | na | 3414 | 0.072 | 0.5401 | Yes |
| 48 | RPL7A | na | 3462 | 0.067 | 0.5411 | Yes |
| 49 | RPL35 | na | 3487 | 0.064 | 0.5437 | Yes |
| 50 | RPS21 | na | 3592 | 0.053 | 0.5389 | No |
| 51 | RPL22L1 | na | 3609 | 0.051 | 0.5413 | No |
| 52 | RPS16 | na | 3626 | 0.051 | 0.5436 | No |
| 53 | RPL18A | na | 3668 | 0.045 | 0.5434 | No |
| 54 | RPS11 | na | 3727 | 0.037 | 0.5413 | No |
| 55 | RPS2 | na | 3817 | 0.025 | 0.5357 | No |
| 56 | RPL10A | na | 3828 | 0.024 | 0.5366 | No |
| 57 | RPL3 | na | 3883 | 0.018 | 0.5334 | No |
| 58 | RPL37 | na | 4179 | -0.015 | 0.5097 | No |
| 59 | RPS27A | na | 4189 | -0.016 | 0.5101 | No |
| 60 | RPL8 | na | 4274 | -0.027 | 0.5050 | No |
| 61 | RPS12 | na | 4369 | -0.037 | 0.4998 | No |
| 62 | RPS3A | na | 4499 | -0.050 | 0.4927 | No |
| 63 | RPL5 | na | 4509 | -0.051 | 0.4956 | No |
| 64 | RPL15 | na | 4832 | -0.084 | 0.4748 | No |
| 65 | RPL12 | na | 4912 | -0.094 | 0.4750 | No |
| 66 | RPL4 | na | 5112 | -0.114 | 0.4666 | No |
| 67 | RPLP0 | na | 5368 | -0.137 | 0.4552 | No |
| 68 | RPS4Y1 | na | 5409 | -0.140 | 0.4621 | No |
| 69 | RPS3 | na | 5595 | -0.158 | 0.4581 | No |
| 70 | RPL17 | na | 7417 | -0.353 | 0.3312 | No |
| 71 | RPS23 | na | 7461 | -0.357 | 0.3536 | No |
| 72 | RPS6 | na | 7644 | -0.376 | 0.3657 | No |
Table: GSEA details [plain text format]

  

Fig 2: KEGG\_RIBOSOME      
 Blue-Pink O' Gram in the Space of the Analyzed GeneSet

  

Fig 3: KEGG\_RIBOSOME: Random ES distribution      
 Gene set null distribution of ES for **KEGG\_RIBOSOME**
